# Les conjonctivites néonatales dans le canton de Glidji au Sud du Togo: une étude transversale à propos de 159 nouveau-nés

**DOI:** 10.11604/pamj.2016.24.42.7623

**Published:** 2016-05-10

**Authors:** Vonor Kokou, Maneh Nidain, Nononsaa Batomguela Kassoula, Fiaty- Amenouvor Kwassi, Banla Meba, Balo Komi Patrice

**Affiliations:** 1 Service d'Ophtalmologie du CHR Tsévié, Lomé, Togo; 2 Service d'Ophtalmologie du CHU Campus, Lomé, Togo; 3 Service d'Ophtalmologie du CHR Sokodé, Lomé, Togo; 4 Service d'Ophtalmologie du CHU Sylvanus Olympio, Lomé, Togo

**Keywords:** Conjonctivite néonatale, staphylocoque aureus, collyre antibiotique, Neonatal conjunctivitis, staphylococcus aureus, antibiotic eye drops, Togo

## Abstract

**Introduction:**

Le but de l’étude était décrire les aspects épidémiologiques des conjonctivites néonatales dans le canton de Glidji au Sud du Togo.

**Methodes:**

Nous avons mené une étude transversale dans les 4 Unités Sanitaires Périphériques du canton de Glidji du 19 Mars au 13 Mai 2009 soit 8 semaines. Tous les nouveau-nés ont été inclus et la conjonctivite néonatale était définie par la présence chez un nouveau-né d'au moins deux des signes suivants: hyperhémie conjonctivale, œdème palpébral, chémosis, sécrétions purulentes, larmoiement. Les paramètres étudiés étaient l’âge, le sexe, les facteurs de risque, les antécédents, la présence ou non de conjonctivite, les germes en causes et l’évolution sous traitement.

**Resultats:**

Sur la période, 159 nouveau-nés ont été examinés. L’âge moyen était de 10,9 jours avec des extrêmes de 0 à 28 jours. Il y avait 80 garçons pour 79 filles soit un sex-ratio de 1,01. Sur les 159 nouveau-nés, 7 cas de conjonctivite ont été diagnostiqués soit une prévalence de 4,4%. Les facteurs de risque identifiés étaient l'accouchement par voie basse et la présence d'IST chez la mère pendant la grossesse. Sur les 7 cas de conjonctivite, l'examen cytobactériologique a permis d'isoler le *staphylococcus aureus* dans 2 cas. L’évolution des cas de conjonctivite sous traitement était favorable avec régression des signes dès le 3è jour.

**Conclusion:**

Les conjonctivites néonatales avaient une prévalence de 4,4% dans le canton de Glidji au sud du Togo et le staphylocoque doré était le germe en cause. Leur prévention passe par un bon suivi lors de la consultation prénatale et l'instillation de collyre antibiotique à la naissance

## Abstract (en)

**Introduction:**

Describe the epidemiology of neonatal conjunctivitis in the Canton of Glidji in southern Togo.

**Methods:**

We conducted a cross-sectional study in 4 Satellite Health Care Units in the Canton of Glidji from 19 March to 13 May 2009 (8 weeks). All newborns were included in the study and neonatal conjunctivitis was defined by the presence in a newborn of at least two of the following signs: conjunctival hyperemia, eyelid swelling, chemosis, purulent discharges, lacrimation. The parameters studied were: age, sex, risk factors, medical history, presence or absence of conjunctivitis, germs responsible for conjunctivitis and evolution under treatment.

**Results:**

During the inclusion period, 159 newborns were examined. The average age was 10.9 days with 0-28 day interval. The study involved 80 boys and 79 girls (sex ratio 1.01). Of the 159 infants, 7 cases of conjunctivitis were diagnosed, representing an overall prevalence of 4.4%. Identified risk factors were: vaginal delivery and the presence of STI during pregnancy. Of the 7 cases of conjunctivitis, cytobacteriological examination allowed the isolation of Staphylococcus aureus in 2 cases. The evolution of the cases of conjunctivitis under treatment was favorable with regression of the signs since the 3rd day.

**Conclusion:**

Neonatal conjunctivitis had a prevalence of 4.4% in the Canton of Glidji in southern Togo and Staphylococcus aureus was the causative germ. Their prevention requires good follow-up prenatal visit and the administration of antibiotic eye drops at birth.

## Introduction

Selon l'Organisation Mondiale de la Santé (OMS), La conjonctivite néonatale, est une inflammation de la conjonctive qui survient au cours des 28 premiers jours de vie [1]. Le Neisseria gonorrhoeae en est le germe le plus en virulent pouvant conduire à la cécité. Dans les pays développés, les conjonctivites néonatales sont devenues rares et bénignes avec l'hygiène et la systématisation des méthodes préventives. A l'opposé, dans les pays économiquement défavorisés, où les conditions d'hygiène et de prophylaxie sont insuffisantes, les conjonctivites néonatales demeurent une menace pour les nouveau-nés [2]. Au Togo, la prévention de la conjonctivite néonatale par la méthode de Crédé par les collyres antibiotiques est répandue mais non systématique. La présente étude a pour but de déterminer les caractéristiques des conjonctivites néonatales en milieu semi-urbain au Sud du Togo.

## Méthodes

Nous avons mené une étude transversale dans les 4 Unités Sanitaires Périphériques du canton de Glidji du 19 Mars au 13 Mai 2009 soit une durée de 8 semaines. Tous les nouveau-nés ont été inclus et la conjonctivite néonatale était définie par la présence chez un nouveau-né d'au moins deux des signes suivants: hyperhémie conjonctivale, œdème palpébral, chémosis, sécrétions purulentes, larmoiement. En cas de conjonctivite, un prélèvement avec examen cytobactériologique des sécrétions était effectué. Les paramètres étudiés étaient l’âge, le sexe, les facteurs de risque, les antécédents, la présence ou non de conjonctivite, les germes en causes et l’évolution sous traitement. La collecte et l'analyse des données ont été effectuées avec le logiciel SPSS 21.

## Résultats

Sur la période, 159 nouveau-nés ont été examinés. La moyenne d’âge était de 10,9 jours avec des extrêmes de 0 à 28 jours. Il y avait 80 garçons pour 79 filles soit un sex-rato de 1,01. En fonction des antécédents, les facteurs de risques identifiés étaient l'accouchement par voie basse dans 157 cas soit 98,7% des cas, la rupture précoce de la poche des eaux dans 34 cas soit 21,4% des cas, l'absence de consultations prénatales dans 34 cas soit 21,4% des cas et la présence de signes d'infections sexuellement transmissibles dans 51 cas soit 31,4%. Les soins oculaires institués à la naissance étaient dominés par l'administration de collyre ou pommade antibiotique dans 125 cas soit 78,6% des cas et de lait maternel dans les yeux dans 25 cas soit 15,7% des cas (Tableau 1). Sur les 159 nouveau-nés, 7 cas de conjonctivite ont été diagnostiqués soit une incidence de 4,4%. Sur ces 7 cas de conjonctivite, aucun soin oculaire à la naissance n'a été institué dans 5 cas, et dans 2 cas le lait maternel a été instillé dans les yeux. Les signes à l'examen ophtalmologique était dominés par l'hyperhémie conjonctivale dans 22 cas soit 13,8% des cas (Tableau 2). Sur les 7 cas de conjonctivite, l'examen cytobactériologique a permis d'isoler le *staphylococcus aureus* dans 2 cas. L’évolution des cas de conjonctivite sous traitement était favorable avec régression des signes dès le 3è jour.

**Tableau 1:** Les soins oculaires institués à la naissance

	Effectif	Pourcentage (%)
Méthode de Crédé classique	1	0,6
Collyre ou Pommade Antibiotique	125	78,6
Lait maternel en collyre	25	15,7
Aucun soin	8	5,1
**Total**	**159**	**100,0**

**Tableau 2:** Les signes de l'examen ophtalmologique

	Effectif	Fréquence (%)
Hyperhémie conjonctivale	22	13,8
Chémosis	5	3,1
Œdème palpébral	4	2,5
Sécrétions purulentes	6	3,8
Pas de signes	124	78

## Discussion

Dans cette étude, l'incidence de la conjonctivite néonatale était de 4,4%. Ayéna et al [2] dans une étude similaire au Togo avait trouvé une incidence de 8% au Nord Togo. L'incidence de la conjonctivite néonatale varie entre 0,5 et 33% d'une étude à l'autre [2–4] et reflète en général le niveau socio-économique de la population. En réalité il existe une sous-estimation de l'incidence de la conjonctivite néonatale liée à la méthode de diagnostic ou à la notification des cas [5]. Les facteurs de risque identifiés dans notre étude étaient l'accouchement par voie basse (98,7%), la rupture précoce de la poche des eaux (21,4%), l'absence de consultations prénatales (44%) et la présence de signes d'infections sexuellement transmissibles (31,4%). Ranjit et al [6]. Dans une étude au Malawi ont montré de leur côtés que les facteurs de risques de conjonctivite néonatale étaient la rupture prématuré des membranes, l'infection maternelle au cours du travail, les infections sexuellement transmissibles, l'accouchement à domicile et l'inhalation de liquide amniotique par le nouveau-né. Le staphylocoque doré était le germe isolé dans notre étude. Dans la littérature, le staphylocoque doré est le germe le plus isolés [3, 7] mais d'autres germes sont aussi en cause notamment le Chlamydia, le gonocoque, l'haemophilus, et le streptocoque [7]. Il est à noter que les techniques microbiologiques standards ne suffisent pas toujours pour isoler certains germes et il faut recourir dans ces cas à la PCR [8]. Les soins oculaires administrés à la naissance étaient les collyres ou pommade antibiotiques dans 78,6% des cas. Mais on a noté aussi l'administration de lait maternel dans les yeux des bébés dans 15,7% des cas. Cette pratique est dangereuse car peut potentiellement aggraver une infection bactérienne et engager le pronostic fonctionnel. Elle doit être découragée et remplacée par la méthode de Crédé. La prévention de la conjonctivite néonatale passe par trois moyens: le dépistage et le traitement des femmes enceintes porteuses d'infections sexuellement transmissibles; la méthode de Crédé avec un collyre à base de nitrate d'argent 1%, ou de collyre antibiotique à tout nouveau-né incluant ceux qui sont nés pas césarienne dans la première heure après la naissance selon la société canadienne de pédiatrie [9]; et par l'hygiène des mains, du corps et des vêtements pour toute personne au contact des nouveau nés.

## Conclusion

Les conjonctivites néonatales avaient une prévalence relativement élevée dans notre étude et le staphylocoque doré était le germe en cause. Leur prévention passe par un bon suivi lors de la consultation prénatale et l'instillation de collyre antibiotique à la naissance. Une sensibilisation de la population est nécessaire pour une adoption de ces bonnes pratiques.
